# Duality of *n*-3 Polyunsaturated Fatty Acids on *Mcp-1* Expression in Vascular Smooth Muscle: A Potential Role of 4-Hydroxy Hexenal

**DOI:** 10.3390/nu7095381

**Published:** 2015-09-21

**Authors:** Kohji Nagayama, Katsutaro Morino, Osamu Sekine, Fumiyuki Nakagawa, Atsushi Ishikado, Hirotaka Iwasaki, Takashi Okada, Masashi Tawa, Daisuke Sato, Takeshi Imamura, Yoshihiko Nishio, Satoshi Ugi, Atsunori Kashiwagi, Tomio Okamura, Hiroshi Maegawa

**Affiliations:** 1 Department of Medicine, Shiga University of Medical Science, Shiga 520-2192, Japan; E-Mails: nagayama@belle.shiga-med.ac.jp (K.N.); sekine@belle.shiga-med.ac.jp (O.S.); f.nakagawa@jclbio.com (F.N.); atsushi.ishikado@jp.sunstar.com (A.I.); hiwasaki@belle.shiga-med.ac.jp (H.I.); tokada@belle.shiga-med.ac.jp (T.O.); dkst0310@belle.shiga-med.ac.jp (D.S.); sugi@belle.shiga-med.ac.jp (S.U.); maegawa@belle.shiga-med.ac.jp (H.M.); 2 Osaka Laboratory, JCL Bioassay Corporation, 5-16-26, Minamisuita, Suita-shi, Osaka 564-0043, Japan; 3 Sunstar Inc., 3-1 Asahi-machi, Takatsuki, Osaka 569-1195, Japan; 4 Joslin Diabetes Centre, Harvard Medical School, MA 02115, USA; 5 Department of Pharmacology, Shiga University of Medical Science, Shiga 520-2192, Japan; E-Mails: tawa@belle.shiga-med.ac.jp (M.T.); timamura@belle.shiga-med.ac.jp (T.I.); okamura@belle.shiga-med.ac.jp (T.O.); 6 Department of Diabetes and Endocrine Medicine, Kagoshima University, Kagoshima 890-8580, Japan; E-Mail: ynishio@m3.kufm.kagoshima-u.ac.jp; 7 Kusatsu General Hospital, 1660, Yabase-cho, Kusatsu, Shiga 525-8585, Japan; E-Mail: kashiwagi@kusatsu-gh.or.jp

**Keywords:** monocyte chemotactic protein 1, 4-hydroxy hexenal, docosahexaenoic acid, eicosapentaenoic acid

## Abstract

*N*-3 polyunsaturated fatty acids such as docosahexaenoic acid (DHA) and eicosapentaenoic acid (EPA) have protective effects against atherosclerosis. Monocyte chemotactic protein (MCP)-1 is a major inflammatory mediator in the progression of atherosclerosis. However, little is known about the regulation of *Mcp-1* by DHA and EPA in vessels and vascular smooth muscle cells (VSMCs). In this study, we compared the effect of DHA and EPA on the expression of *Mcp-1* in rat arterial strips and rat VSMCs. DHA, but not EPA, suppressed *Mcp-1* expression in arterial strips. Furthermore, DHA generated 4-hydroxy hexenal (4-HHE), an end product of *n*-3 polyunsaturated fatty acids (PUFAs), in arterial strips as measured by liquid chromatography-tandem mass spectrometry. In addition, 4-HHE treatment suppressed *Mcp-1* expression in arterial strips, suggesting 4-HHE derived from DHA may be involved in the mechanism of this phenomenon. In contrast, *Mcp-1* expression was stimulated by DHA, EPA and 4-HHE through p38 kinase and the Keap1-Nuclear factor erythroid-derived 2-like 2 (Nrf2) pathway in VSMCs. In conclusion, there is a dual effect of *n*-3 PUFAs on the regulation of *Mcp-1* expression. Further study is necessary to elucidate the pathological role of this phenomenon.

## 1. Introduction

Atherosclerosis is characterized by accumulation of oxidized fat, thickening of vessel walls by collagens secreted by proliferating vascular smooth muscle cells, and macrophage filtration [1]. There are several steps in the progression of atherosclerosis: (1) endothelial dysfunction, (2) migration of leukocytes and smooth muscle cells into the vessel wall, (3) foam cell formation, and (4) degradation of extracellular matrix. Epidemiologically, fish consumption negatively correlates with cardiovascular events, suggesting beneficial effects of *n*-3 polyunsaturated fatty acids (PUFA) [2,3]. Other clinical studies have indicated that *n*-3 PUFAs improved the carotid intima-media thickness and endothelial function [4,5], suggesting that *n*-3 PUFAs attenuate atherosclerosis by decreasing migration of leukocytes and proliferation of smooth muscle cells. This is supported by animal experiments that showed *n*-3 PUFAs attenuated VCAM-1 expression and macrophage filtration [6]. *N*-3 PUFAs mainly consist of eicosapentaenoic acid (EPA) and docosahexaenoic acid (DHA). However, the difference between EPA and DHA in terms of their anti-atherosclerotic effect is still unclear.

Monocyte chemotactic protein (MCP)-1/chemokine (C-C motif) ligand 2 (CCL2) is expressed in inflammatory cells and stromal cells such as endothelial and smooth muscle cells, and its expression is regulated by proinflammatory stimuli and tissue injury. *Mcp-1* is regulated both by the nuclear factor kappa-light-chain-enhancer of activated B cells (NF-κB) pathway and stress-activated kinases including p38, ERK and JNK [7]. There are several potential mechanisms that explain the anti-inflammatory effect of EPA and DHA. A recent report revealed that G-protein coupled receptor 120 (GPR120) is a receptor for DHA that mediates anti-inflammatory and insulin-sensitizing effects in rodents [8]. Other reports have suggested that resolvins and protectins—which are derived from EPA and DHA—are mediators of the anti-inflammatory effects [9]. We have recently reported that 4-hydroxy hexenal (4-HHE)—an end product of *n*-3 PUFA peroxidation—activates the nuclear factor erythroid 2-related factor 2 (Nrf2)-Kelch-like ECH-associated protein 1 (Keap1) pathway in human umbilical vein endothelial cells (HUVECs), contributing to endothelial function and antioxidative activity [10,11].

Nrf2 is a redox-sensitive master regulatory transcription factor regulated by Keap1. Electrophiles, shear stress, and reactive oxygen species (ROS) stimulate modification of the cysteine residues of Keap1, which allows its translocation to the nucleus. Nrf2 induces antioxidant enzymes such as heme oxygenase-1 (Hmox1) through the antioxidant response element (ARE) consensus sequence [12,13]. The 4-HHE induces Nrf2-mediated Hmox1 expression in multiple organs [14,15]. In addition, it has also been reported that DHA induces Nrf2-mediated Hmox1 expression in human vascular smooth muscle cells (VSMCs) isolated from small pulmonary artery or endothelial cells [16,17].

Therefore, we examined the regulation of *Mcp-1* by DHA and EPA in arterial strips and VSMCs. Furthermore, we measured the 4-HHE content by a liquid chromatography-tandem mass spectrometry (LC-MS/MS) and tested its role in these tissues.

## 2. Methods

### 2.1. Reagents

Dulbecco’s Modified Eagle’s Medium (DMEM) and fetal bovine serum (FBS) were obtained from Life Technologies (Grand Island, NY, USA). EPA, DHA, and 4-HHE were purchased from Cayman (Ann Arbor, MI, USA). The MTT assay kit, anti-β-actin (A5316) antibody and *N*-acetyl-l-cysteine were purchased from Sigma-Aldrich (St. Louis, MO, USA). Fatty acid-free bovine serum albumin (BSA) was purchased from Nacalai Tesque (Kyoto, Japan). Anti-p38 (#9112), anti-phospho-p38 (#9211), anti-ERK1/2 (#9102), anti-phospho-ERK1/2 (#9106), anti-JNK (#9252), anti-phospho-JNK (#9251), and anti-caspase-3 (#9661) antibodies were purchased from Cell Signaling (Danvers, MA, USA). Horseradish peroxidase-linked anti-mouse and anti-rabbit antibodies were purchased from Amersham Biosciences Corp. (Piscataway, NJ, USA). 2′7′-Dichlorodihydrofluorescein diacetate (H_2_DCFDA) and small interfering RNA (SiRNA) reagents were purchased from Life Technologies (Tokyo, Japan). SB203580, PD98059 and SP600125 were purchased from Calbiochem (Cambridge, UK).

### 2.2. Animals and Experimental Procedures

All animal experimentation was approved by the committee for Animal Research of Shiga University of Medical Science (No. 2014-4-8, 7 May 2014). The experimental procedure for artery strips was performed as previously reported [18]. Briefly, eight-week-old male Sprague-Dawley rats (Japan SLC, Shizuoka, Japan) were housed in an environmentally controlled room with a 12 h light/dark cycle and free access to food and water. Rats were fed a regular diet (Dyets Inc., Bethlehem, PA, USA) for 12 weeks. After 12 h of fasting, rats were sacrificed by bleeding from the abdominal aorta under deep anesthesia. The thoracic aorta was dissected, excised, and cut into strips with special care being taken to preserve the endothelium. The strips were then fixed vertically between hooks in a muscle bath (10-mL capacity) containing modified Ringer-Locke solution bubbled with a gas mixture of 95% O_2_ and 5% CO_2_, pH 7.4 at 37 ± 0.3 °C. After treatment with DHA, EPA or 4-HHE for 6 h, the arterial strips were immediately freeze-clamped by liquid nitrogen, and stored at −80 °C. For lipid extraction, the frozen tissues were pulverized into a fine powder using a Cryo Press disruptor (Microtec Co., Ltd., Chiba, Japan). This fine powder was weighed on an ME235 electronic balance (Sartorius AG, Göttingen, Germany), homogenized in 490 µL of chloroform/methanol (1:1, v/v) and 10 μL of dibutylhydroxytoluene solution (10 mg/mL in ethanol), and incubated at 36 °C for 1 h [19]. The resulting solution was used for measuring the 4-HHE content.

### 2.3. Cell Culture

VSMCs were isolated from the aortas of male Sprague-Dawley rats (150–200 g) by enzymatic digestion as previously described [20]. Briefly, cells were maintained in DMEM supplemented with 10% FBS, and used between the 4th–12th passages except for primary cells, showing a dramatic growth rate because of transformation. Cells were grown to confluence in 12-well plates, and cell growth was arrested for 24 h in DMEM supplemented with 1% FBS before the real-time quantitative polymerase chain reaction (RT-qPCR) experiments.

### 2.4. Fatty Acid Treatment

DHA or EPA was administered as a complex with fatty acid-free BSA as previously described [15]. Briefly, 0.3 mM DHA or EPA was dissolved in ethanol (2.5 mL), and gradually solubilized in an 8.4% BSA solution (14.3 mL) at 37 °C. The 4-HHE was dissolved in dimethyl sulfoxide and then in serum-containing medium.

### 2.5. Messenger RNA (mRNA) Extraction and Real-Time RT-qPCR Analysis

Total RNA was extracted from cells and tissues using a Total RNA Mini Kit (Bio-Rad, Hercules, CA, USA). Single-stranded cDNA was synthesized from 1.5 μg of total RNA using the Prime Script RT Reagent Kit (Takara Bio, Shiga, Japan), and endogenous genomic DNA was degraded by DNase I (Life Technologies, CA, USA). RT-qPCR experiments were carried out with SYBR Green PCR master mix (Life Technologies, CA, USA) and the ABI 7500 Fast Real-Time PCR System (Applied Biosystems, Foster City, CA, USA). All the quantitative data were normalized against the expression levels of 18S rRNA (18S). RT-qPCR conditions were 95 °C for 10 min, followed by 40 cycles of 95 °C for 15 s and 60 °C for 1 min. The primers for the RT-qPCR are listed in Table 1.

**Table 1:** Candidate genes, primer sequences and accession numbers.

	Forward Primer	Reverse Primer	Accession Number
Mcp-1	GCTGCTACTCATTCACTGGCAA	TGCTGCTGGTGATTCTCTTGTA	NM_031530.1
Hmox-1	TCTATCGTGCTCGCATGAAC	AAGGCGGTCTTAGCCTCTTC	NM_012580.2
Nrf2	GGAGCAATTCAACGAAGCTC	ACAGTTCTGAGCGGCAACTT	NM_031789.2
18S	TTCCGATAACGAACGAGACTCT	TGGCTGAACGCCACTTGTC	NR_046237.1

### 2.6. Quantitative Analysis of 4-HHE in Biological Samples

The 4-HHE in aorta and VSMCs was quantitatively analyzed using LC-MS/MS procedure as described previously [15,21]. Briefly, a standard solution of 4-HHE (Cayman Chemical Co., Ann Arbor, MI, USA) was used for the calibration curve. Solid-phase extraction was done using a mixed-mode anion exchange solid-phase extraction (SPE) cartridge (Oasis MAX, Waters, Milford, MA, USA). An ACQUITY CSH C18 column (Waters) was used for separating 4-HHE. Electrospray ionization (ESI) was carried out with API4000 operating in the positive ionization and SRM mode. The SRM transitions for CHD-derivatized 4-HHE were *m*/*z* 284-216.

### 2.7. MTT Assay for Cell Viability

Rat VSMCs were seeded on 24-well plates. To determine the cell toxicity of DHA, EPA and 4-HHE, confluent cells were exposed to these reagents for 24 h, and then washed with phosphate-buffered saline (PBS). Cell viability was determined by the conventional MTT assay as previously described [11]. The absorbance of BSA-treated cells was used as the control.

### 2.8. Reactive Oxygen Species (ROS) Measurement Assay

Intracellular ROS production was determined using the fluorescent probe H_2_DCFDA in VSMCs incubated with 20 µM H_2_DCFDA for 20 min as previously described [11]. Following washing with PBS, cells were incubated with 50 µM DHA or 50 µM EPA. The fluorescence emitted from the cells was recorded immediately at 492 nm (excitation) and 525 nm (emission) using a fluorescent microplate reader (Tecan, Männedorf, Switzerland) over a 2-h period.

### 2.9. Western Blot Analysis

Total protein samples from VSMCs were prepared as previously descried [11], and were resolved by SDS-PAGE before being transferred to PVDF membranes. Membranes were incubated with antibodies against p38, ERK, JNK, their phosphorylated forms, caspase-3, or β-actin. Blots were then incubated with horseradish peroxidase-linked second antibody (Amersham, Buckinghamshire, UK), followed by chemiluminescence detection (PerkinElmer, Waltham, MA, USA).

### 2.10. Statistical Analysis

Data are presented as mean ± SE, unless otherwise stated. Differences between more than three groups were analyzed by Tukey–Kramer test. When two groups were compared, differences were analyzed by two-tailed Student’s *t*-test. *P* < 0.05 was considered statistically significant.

## 3. Results

### 3.1. Docosahexaenoic Acid (DHA)—Though Not Eicosapentaenoic Acid (EPA)—Inhibits Mcp-1 mRNA Expression in Rat Aorta

**Figure 1:**
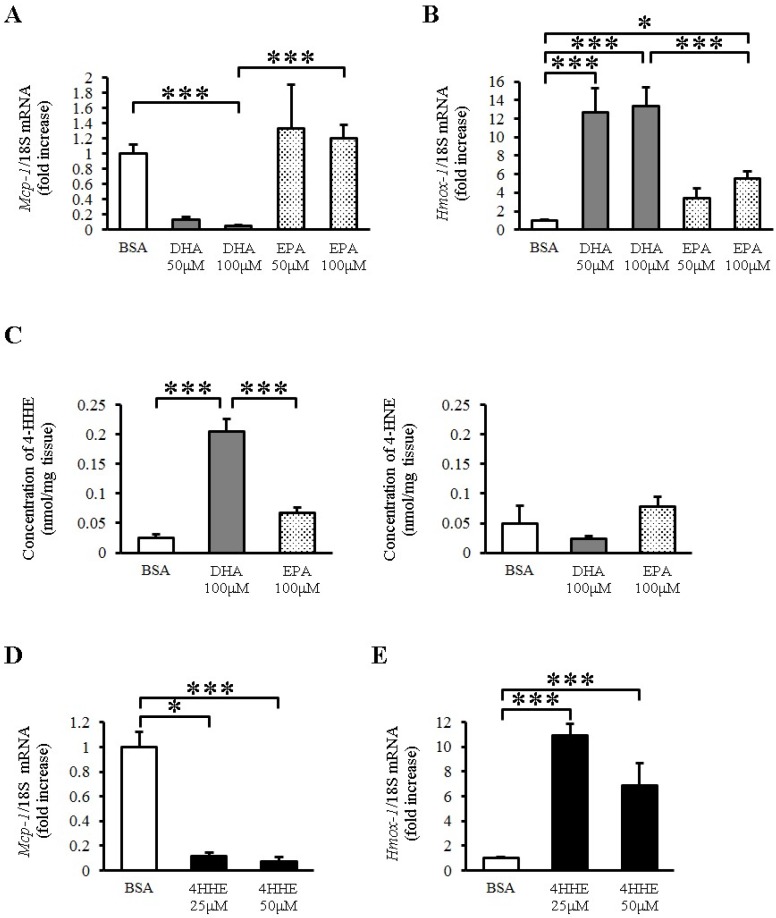
Docosahexaenoic acid (DHA)-derived DHA generated 4-hydroxy hexenal (4-HHE) inhibits the expression of *Mcp-1* Messenger RNA (mRNA), but induces *heme oxygenase 1* (*Hmox-1*) mRNA in rat aorta. Rat arterial strips were treated with bovine serum albumin (BSA), DHA (50–100 μM), EPA (50–100 μM) or 4-HHE (25–50 μM) for 6 h under *ex vivo* conditions. (**A**,**B**) Relative mRNA expression of *Mcp-1* (**A**) and *Hmox-1* (**B**) in arterial strips was quantitated using the real-time quantitative polymerase chain reaction (RT-qPCR). Results were normalized against 18S rRNA and expressed as fold increase over control. (**C**) 4-HHE and 4-HNE content were measured by a liquid chromatography-tandem mass spectrometry (LC-MS/MS). (**D**,**E**) Relative mRNA expression of *Mcp-1* (**D**) and *Hmox-1* (**E**) in arterial strips was quantitated using RT-qPCR. Results were normalized as above. Results are expressed as mean ± SE of 4–8 animals (*n* = 3–22; **A**,**B**,**D**,**E**), or a single experiment (*n* = 3; **C**). * *P* < 0.05, *** *P* < 0.001, compared with BSA control. NS, no significant difference.

To explore the direct effects of EPA and DHA on vessels, we examined the expression of *Mcp-1* mRNA in rat arterial strips. DHA (50–100 μM) but not EPA (50–100 μM) almost completely inhibited the expression of *Mcp-1* mRNA compared with BSA (Figure 1A). In contrast, DHA increased the expression of *heme oxygenase 1 (Hmox-1)* (Figure 1B), which is a known antioxidative gene in vessels. EPA also increased the expression of *Hmox-1*, but to a lesser extent than DHA did (Figure 1B). Because *Hmox-1* is a target gene of the Keap1-Nrf2 pathway, we measured the lipid peroxidation product levels in rat arterial strips by LC-MS/MS with or without *n*-3 PUFA incubation. We found that DHA but not EPA increased the tissue 4-HHE content, whereas it did not change the content of 4-hydroxy 2-noneral (4-HNE), a lipid peroxidation product derived from *n*-6 PUFA (Figure 1C). To test the role of 4-HHE, we exposed the arterial strips to 4-HHE and found that it inhibited the expression of *Mcp-1* (Figure 1D) and increased that of *Hmox-1* (Figure 1E) in rat aortic strips, suggesting that DHA regulates *Mcp-1* and *Hmox-1* expression through 4-HHE.

### 3.2. Paradoxical Increase in Mcp-1 by DHA, EPA and 4-HHE in VSMCs

**Figure 2:**
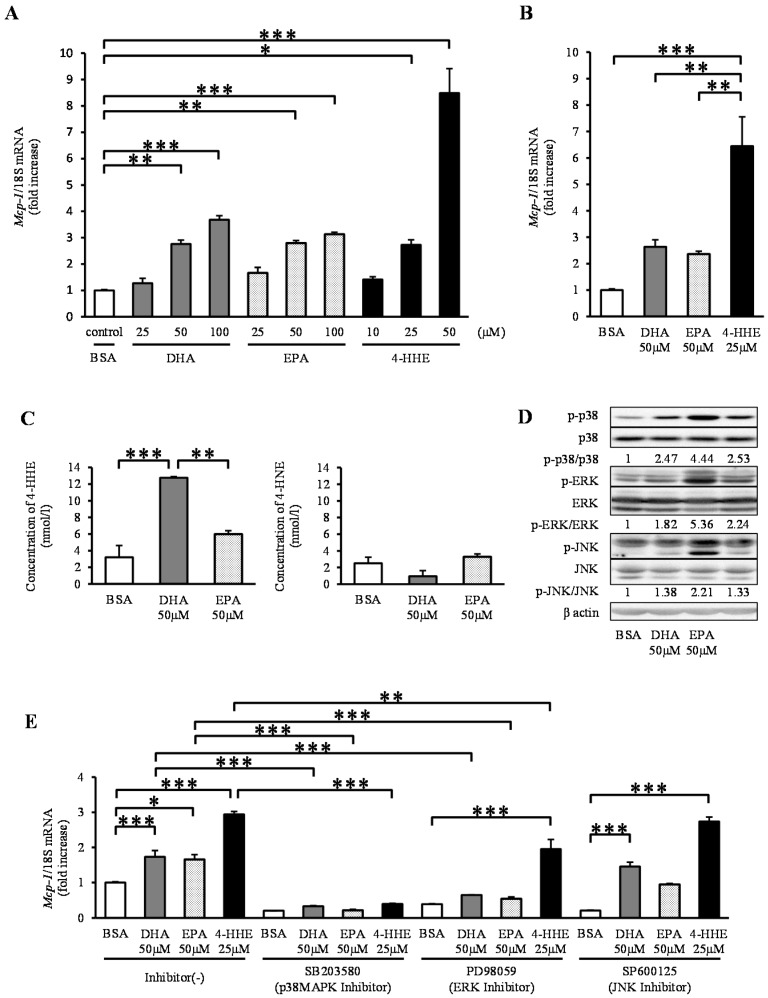
DHA, EPA, and 4-HHE induce *Mcp-1* expression through the p38 mitogen-activated protein kinase (MAPK) pathway in VSMCs. VSMCs (Passage 4–12) were treated with the indicated reagent for 6 h (**A**). (**B**) Primary vessels and vascular smooth muscle cells (VSMCs) (Passage 1) were treated with BSA, DHA (50 μM), EPA (50 μM) or 4-HHE (25 μM) for 6 h. Relative mRNA expression of *Mcp-1* was quantitated using RT-qPCR. The results were normalized against 18S rRNA and expressed as fold increase over control. (**C**) 4-HHE and 4-HNE content in VSMCs were measured using LC-MS/MS. (**D**) p38, ERK, JNK and their phosphorylated forms, and β-actin were determined by Western blotting. DHA (50 μM), EPA (50 μM) or 4-HHE (25 μM) were added for 10 min. (**E**) Pretreatment with p38 kinase inhibitor (SB203580; 10 μM), ERK inhibitor (PD98059; 25 μM) or JNK inhibitor (SP600125; 10 μM) was performed for 30 min before BSA, DHA, EPA or 4-HHE incubation. The results were normalized against 18S rRNA and expressed as fold increase over corresponding control. (**A**) Values represent the mean ± SE of four independent experiments (*n* = 9); (**B**) a single experiment (*n* = 3); (**C**) a single experiment (*n* = 3); or (**E**) three independent experiments (*n* = 3–9). * *P* < 0.05, ** *P* < 0.01, *** *P* < 0.001, compared with corresponding control.

In contrast to the results observed for arterial strips, DHA, EPA and 4-HHE increased the expression of *Mcp-1* mRNA in a dose-dependent manner in rat VSMCs (Figure 2A). To clarify the differences in *Mcp-1* responses between rat arterial strips and VSMCs (Passage 4–12), we performed the same experiment using primary VSMCs (Passage 1). Similar to VSMCs (Passage 4–12), DHA, EPA, and 4-HHE increased the expression of *Mcp-1* in primary VSMCs (Figure 2B). Similar to rat arterial strips, DHA (50 μM), but not EPA (50 μM), increased the content of 4-HHE in VSMCs (Figure 2C), whereas it did not change the 4-HNE content.

Because *n*-3 PUFAs are known activators of the mitogen-activated protein kinase (MAPK) family, we assessed the phosphorylation levels of p38 kinase, ERK and JNK. DHA, EPA and 4-HHE increased the phosphorylation levels of p38, ERK and JNK (Figure 2D). To understand the effect of the MAPK family on the *Mcp-1* expression, we tested the effect of MAPK inhibitors on DHA-, EPA- or 4-HHE-induced *Mcp-1* expression. Pre-incubation with the p38 kinase inhibitor SB203580 completely suppressed the induction of *Mcp-1* expression (Figure 2E). The ERK inhibitor PD98059 had a partial inhibitory effect on *Mcp-1* expression, whereas the JNK inhibitor SP600125 did not (Figure 2E).

### 3.3. 4-HHE Derived from DHA Induces Mcp-1 Expression through the Nrf2 Pathway in Human Vascular Smooth Muscle Cells (VSMCs)

**Figure 3:**
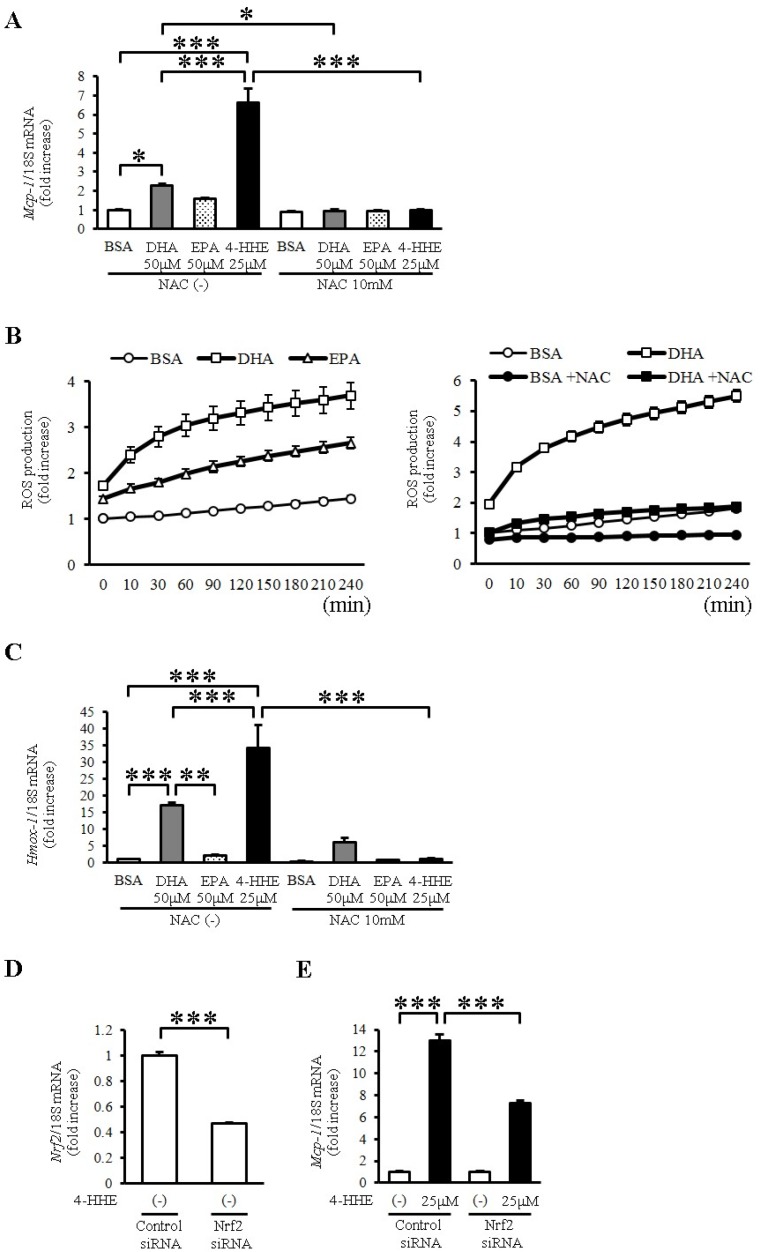
DHA-derived 4-HHE induces *Mcp-1* expression partially through the oxidative stress-induced Nrf2 pathway in VSMCs. (**A**,**C**) VSMCs were treated with *N*-acetyl-l-cysteine (NAC; 10 mM) for 1 h before incubation with BSA, DHA (50 μM), EPA (50 μM) or 4-HHE (25 μM) for 6 h. Relative mRNA expression of *Mcp-1* (**A**) and *Hmox-1* (**C**) in VSMCs was quantitated using RT-qPCR. Results were normalized against 18S rRNA and expressed as fold increase over control. (**B**) Reactive oxygen species (ROS) production was measured by 2′7′-Dichlorodihydrofluorescein diacetate (H_2_DCFDA). BSA, DHA (50 μM) or EPA (50 μM) was added for 4 h (left panel). BSA or DHA (50 μM) was added with or without NAC (10 mM) for 4 h (right panel). (**D**,**E**) VSMCs were treated with *Nrf2* siRNA (40 nM) or control siRNA (40 nM). After 24 h, VSMCs were treated with vehicle or 4-HHE (25 μM) for 6 h. Relative mRNA of *Nrf2* (**D**) and *Mcp-1* (**E**) was quantitated using RT-qPCR. Values represent the mean ± SE of three independent experiments (*n* = 9; **A**,**C**); a single experiment (*n* = 3; **B**); and two independent experiments (*n* = 6; **D**,**E**). * *P* < 0.05, *** *P* < 0.001, compared with the corresponding control.

To evaluate the oxidative stress induced by DHA and 4-HHE, we used *N*-acetyl-l-cysteine (NAC), a known antioxidant that mimics glutathione. Pretreatment with NAC (10 mM) completely inhibited the DHA-, EPA- and 4-HHE-induced *Mcp-1* expression (Figure 3A). Furthermore, DHA and EPA increased ROS production measured by H_2_DCFDA (Figure 3B). NAC pretreatment completely inhibited the DHA-induced ROS production in VSMCs (Figure 3B), supporting the role of oxidative stress in the DHA-induced *Mcp-1* expression.

To evaluate Nrf2 activation by DHA and 4-HHE, we examined the mRNA expression of *Hmox1*, a target of Nrf2, in VSMCs. We found that DHA and 4-HHE stimulated the expression of *Hmox1* mRNA in VSMCs, and that NAC inhibited the DHA- and 4-HHE-induced *Hmox-1* expression (Figure 3C). As expected, the 4-HHE-induced *Mcp-1* expression was decreased by siRNA against *Nrf2* (Figure 3D,E).

### 3.4. DHA Induces Apoptosis of VSMCs through 4-HHE

**Figure 4:**
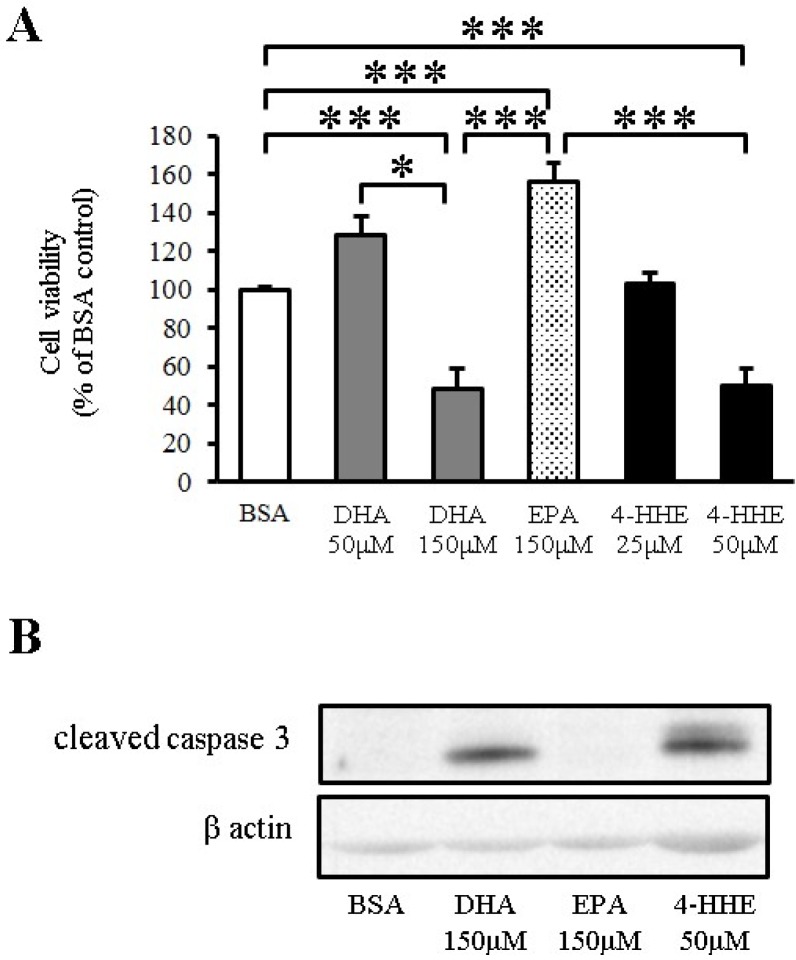
DHA-derived 4-HHE reduces cell viability through apoptosis in VSMCs. (**A**) VSMCs were treated with a high concentration of DHA (150 μM), EPA (150 μM) or 4-HHE (50 μM) for 24 h. Cell viability was determined by the MTT assay. Values are expressed as percentage of cell survival, and each value represents the mean ± SE of five experiments (*n* = 15). (**B**) Cleaved caspase-3 and β-actin were determined by Western blotting. DHA (150 μM), EPA (150 μM) or 4-HHE (50 μM) were added for 6 h. * *P* <0.05, *** *P* < 0.001, compared with BSA control.

To test the toxicity of *n*-3 PUFA in VSMCs, VSMCs were incubated for 24 h with DHA, EPA or 4-HHE at a higher but physiological concentration, followed by measurement of cell viability by the MTT assay. DHA and 4-HHE only decreased cell viability at a high concentration (150 μM) compared with the BSA control (Figure 4A). In contrast, EPA did not decrease the cell viability of VSMCs (Figure 4A).

Apoptosis is a known downstream process of ROS production. Increased cleaved caspase-3 expression measured by Western blot analysis indicated that the cell toxicity of DHA and 4-HHE was caused by the induction of apoptosis (Figure 4B).

## 4. Discussion

Our study has three important findings. First, DHA and 4-HHE, but not EPA, inhibited *Mcp-1* expression in rat arterial strips. Second, DHA and 4-HHE inhibited cell survival by promoting apoptosis in VSMCs. Third, DHA, EPA and 4-HHE stimulated *Mcp-1* expression via oxidative stress, p38 and the Keap1-Nrf2 pathway in VSMCs.

DHA and 4-HHE, but not EPA, inhibited *Mcp-1* expression in rat arterial strips. Previous studies have shown that *n*-3 PUFAs affect inflammation and plaque stability [22,23], which is consistent with the inhibitory effect of DHA on *Mcp-1* expression under *ex vivo* conditions in our study. In contrast, EPA had almost no effect on *Mcp-1* expression (Figure 1A). We assume this difference was the result of Nrf2 activation by 4-HHE, because we observed a similar difference between DHA and EPA in HUVECs, which was explained by the generation of 4-HHE [10,11,14,15,16]. As expected, DHA preferentially increased intracellular 4-HHE content in rat arterial strips compared with EPA (Figure 1C). In addition, 4-HHE directly inhibited *Mcp-1* expression in rat arterial strips (Figure 1D). Although the molecular mechanism underlying this phenomenon is not clear, 4-HHE may be a mediator of the anti-inflammatory effect of DHA.

We also found that DHA and 4-HHE at a higher concentration and longer incubation inhibited cell survival by promoting apoptosis in VSMCs. In agreement with our study, previous reports have shown that DHA induced apoptosis in VSMCs or cancer cells through p38 MAPK activation at 24 hours [24,25]. Another report demonstrated that 4-HHE induced cytotoxic and negative effects on YPEN-1 prostatic endothelial cells at 24 hours [26]. Because migration of transformed VSMCs is one of the main features of atherosclerosis [27], 4-HHE-induced apoptosis, followed by macrophage clearance via *Mcp-1* expression may be beneficial. Conversely, apoptosis in advanced plaque lesions may be detrimental. This discrepancy might explain the inconsistent effects of *n*-3 PUFAs on cardiovascular events in a secondary prevention study [28].

Both DHA and EPA stimulated *Mcp-1* expression in VSMCs. Our preliminary data suggest that other fatty acids including palmitic and arachidonic acid also stimulate *Mcp-1* expression. We speculate that this was due to oxidative stress induced by fatty acids—known as lipotoxicity [29,30]—rather than being an *n*-3 PUFA-specific effect. In addition to lipotoxicity, DHA preferentially degrades to 4-HHE via peroxidation. 4-HHE has an aldehyde residue that causes a Michael reaction with proteins, forming protein adducts [13]. *N*-acetyl-l-cysteine—a known antioxidant—protected VSMCs from 4-HHE-induced Nrf2 action through the formation of 4-HHE-NAC adducts. *Nrf2* siRNA inhibited the 4-HHE-induced *Mcp-1* expression, suggesting that DHA stimulated *Mcp-1—*at least in part—through the 4-HHE-Nrf2 pathway.

The opposite effects caused by DHA on *Mcp-1* mRNA expression between arterial strips and VSMCs were observed in this study. VSMCs were cultured in a different environment as compared to arterial strips: culture media, growth factors, and monolayer. These biological factors may explain the discrepancy between arterial strips and VSMCs. Other possibilities are that endothelial cells are a major source of *Mcp-1* mRNA and that endothelial cells induce smooth muscle cells to suppress *Mcp-1* mRNA in response to DHA. To test this possibility, DHA-induced *Mcp-1* expression were analyzed in VSMCs with the condition media from rat aortic endothelial cells, and aortic strips without endothelial cells. Our preliminary data suggest that DHA-induced *Mcp-1* is not affected by endothelial cells.

DHA but not EPA produces 4-HHE in rat arterial strips and VSMCs. Previous reports from our group and others have shown that the 4-HHE content or 4-HHE adducts increased after fish oil treatment in heart, liver and other tissues [15,31,32]. Furthermore, previous reports have shown that plasma 4-HHE levels increased following supplementation with DHA or fish-based diet intervention in humans [33,34]. The reason for the difference in 4-HHE generation between DHA and EPA is not clear; hence, further experiments are necessary to elucidate this phenomenon.

Concentrations of EPA and DHA (25–150 μM) used in this study are similar to previous studies [3,11,14,16]. These concentrations are relatively low compared to the reported concentrations in human plasma (200–400 μM) [3]. As shown in Figure 4, high concentrations of DHA had a cytotoxic effect compared to high concentrations of EPA. This phenomenon was consistent with a previous study that showed DHA but not EPA had a profound growth inhibitory effect on HPV16 immortalized cells but not on normal cells [35]. In addition, DHA has strong inhibitory effects on multiple cancer cell lines [26]. We speculated that 4-HHE preferentially generated by DHA might explain the difference between DHA and EPA.

There were some limitations in this study. First, we could not identify the molecular mechanism of the DHA-induced *Mcp-1* decrease in artery strips, although our data suggest that 4-HHE-induced Nrf2 activation may play a role. Second, the 4-HHE content measured was free 4-HHE. Because 4-HHE generates 4-HHE adducts—especially with glutathione—the total 4-HHE content in the tissues may be higher. We incubated VSMCs with 4-HHE at 25 μM based on its ability to stimulate Hmox1. Third, the clinical significance of the DHA-induced *Mcp-1* expression is still not clear.

## 5. Conclusions

DHA had contrasting effects on *Mcp-1* expression in vessels and VSMCs. We suggest a possible role for Nrf2 activation by DHA-derived 4-HHE. Furthermore, 4-HHE derived from DHA decreased cell viability by inducing apoptosis in VSMCs. These findings may explain the different effects of EPA and DHA on vessels.

## Abbreviations

Monocyte chemotactic protein 1 (Mcp-1), nuclear factor erythroid 2-related factor 2 (Nrf2), 4-hydroxy hexenal (4-HHE), polyunsaturated fatty acids (PUFAs), docosahexaenoic acid (DHA), eicosapentaenoic acid (EPA), heme oxygenase-1 (Hmox1), vascular cell adhesion protein 1 (VCAM-1), extracellular signal-regulated kinase (ERK), c-JUN N-terminal kinase (JNK), p38 mitogen-activated protein kinase (p38).
